# A biomechanical experiment and clinical study of the use of figure of eight plus circular wiring fixation for the treatment of olecranon fractures

**DOI:** 10.3892/etm.2012.731

**Published:** 2012-09-01

**Authors:** WULIAN WANG, GUANGWEN WU, FUER SHEN, YIYUAN ZHANG, XIANXIANG LIU

**Affiliations:** 1 Academy of Integrative Medicine, Fujian University of Traditional Chinese Medicine, Fuzhou 350122;; 2 Department of Orthopedics, the Fuzhou No. 2 Hospital, Fuzhou 350007;; 3 Department of Management, Fujian University of Traditional Chinese Medicine, Fuzhou 350122, P.R. China

**Keywords:** figure of eight plus circular wiring, olecranon fractures, internal fixation, biomechanics

## Abstract

The aim of the present study was to evaluate the stability of the figure of eight plus circular wiring fixation technique compared with four common internal fixation techniques and to provide experimental data for the selection of internal fixation techniques clinically. A total of 20 fresh cadaveric elbow joints were used as transverse, oblique and comminuted olecranon fracture models. Five techniques of internal fixation were investigated: circular wiring, figure of eight wiring, circular plus figure of eight wiring, Kirschner wire (K-wire) and screw fixation. The elbow joints were flexed at 90°. The fixation performance was tested using a high-precision displacement sensor. Displacement-load curves revealed that the strength of internal fixation was weakest when using circular wiring alone and that circular wiring plus figure of eight wiring fixation was stronger than that of figure of eight wiring or screw fixation. The difference was statistically significant (P<0.05). There were no significant differences between circular wiring plus figure of eight wiring fixation and K-wire fixation in the transverse and oblique fracture models (P>0.05). However, figure of eight plus circular wiring fixation was superior to K-wire fixation in the comminuted fracture model, with a tensile force of 67.42±2.17 vs. 58.52±2.17 N, respectively (P<0.05). All 152 patients with olecranon fractures who received circular wiring plus figure of eight wiring fixation recovered and 108 were included in the follow-up for an average of 12 months. The rate of excellent/fairly good recovery was 98.10%. Due to its reliability, simple surgery, lower invasiveness and lower cost, figure of eight plus circular wiring fixation is an ideal choice for the internal fixation of olecranon fractures, particularly comminuted fractures, compared with circular wiring, figure of eight wiring or screw fixation.

## Introduction

Olecranon fractures are among the most common traumas of the elbow joint, representing approximately 10% of all fractures in the proximity the elbow (1). In addition to direct trauma, overloading of the triceps muscle may also cause a fracture (1,2). Fractures due to direct trauma are usually comminuted fractures which impact into the interior of the distal humerus (2). With the exception of certain avulsion fractures, the majority of olecranon fractures involve the articular surface. When an olecranon fracture occurs, its normal anatomical association is damaged and the biomechanical balance is disrupted. Due to the proximal traction of the triceps brachii and counteraction of the trochlea of the humerus, pressure is generated on the olecranon and tension occurs in the cortex. Thus, the fractured end has a tendency to separate. An olecranon fracture is a type of internal joint fracture which, if not properly treated, may result in fracture nonunion, synarthrophysis, myositis ossificans, articular instability, traumatic arthritis and delayed paralysis of the cubital nerve (3). Therefore, three criteria should be met in the treatment of olecranon fractures (4): i) anatomical reduction and the restoration of smooth articular surfaces; ii) firm fixation to allow positive and non-intensive functional training to begin prior to the confirmation of complete healing by X-ray; and iii) early-stage functional training to restore the function of the elbow joint. Olecranon fractures are usually transverse or oblique. However, with the intensification of external forces, the articular surface of the olecranon may be comminuted and crushed in the center or an avulsion fracture of the coronoid process may occur (5). Open reduction internal fixation is the basic method for treating olecranon fractures.

There are a number of internal fixation methods and types of equipment for treating olecranon fractures. Halling *et al* (6) advocated the tension band technique while Assom *et al* (7) applied the thread fixation technique. However, each fixation technique has its advantages and disadvantages. For example, although tension-band wiring with Kirschner wires is widely used in the surgical treatment of olecranon fractures, particularly in non-comminuted fractures, and may provide a stable construct to allow early joint motion (1,8,9), it may result in tenderness at pin sites at later stages (10). In comminuted fractures, particularly in cases with bone loss, initiating early movements following tension-band wiring may cause problems (11). The use of tension-band wiring in comminuted fractures may also cause contractions in the sigmoid notch (12). In the biomechanical study by Fyfe *et al* (13), adequate rigidity was ensured by using tension-band wiring in models with transverse osteotomies but a significantly more stable fixation was achieved using plate fixation in comminuted osteotomies. Therefore, it is essential to identify a convenient, reliable, less invasive, less costly and practical internal fixation method.

**Figure 1:**
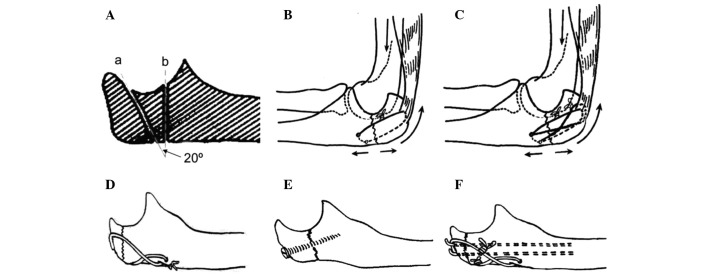
Schematic diagram of fracture model and different wiring methods. (A) Olecranon fracture model: (a) oblique fracture model, (b) transverse fracture model and (a+b) comminuted fracture model which combined the two models. (B) Circular wiring internal fixation. (C) Figure of eight plus circular wiring internal fixation. (D) Figure of eight wiring. (E) Screw fixation. (F) Kirschner tension band wiring.

Since 1987, the Department of Orthopedics (the Fuzhou No. 2 Hospital, Fuzhou, China) have practiced improved internal fixation by wiring, i.e., using figure of eight plus circular wiring fixation to treat olecranon fractures, with satisfactory clinical results. This method has an additional circular wire to the figure of eight wire (Fig. 1C), thus the fracture stability and the tension band effect of the wire are reinforced. This conforms to the concept and principles of the Association for the Study of Internal Fixation (ASIF), as well as the tension band and flexible fixation principles. It may be applied to all types of fractures, providing firm fixation and permitting early-stage functional training. It appears that by using this method, fracture healing is faster and has a greater therapeutic effect. However, this technique lacks quantitative indices and experimental data to verify its suitability for treating olecranon fractures. In the present study, an experiment was designed for the biomechanical comparison of this method with four others: circular wiring, figure of eight wiring, screw fixation and Kirschner wire tension band fixation. The fixation stability of this method for treating olecranon fractures was studied, clinical cases were collected and the efficacy of the treatment was observed. The results of the present study demonstrated that figure of eight plus circular wiring fixation is a convenient, reliable, less invasive, less costly and practical internal fixation method.

## Materials and methods

### 

#### Model establishment

A total of 20 fresh cadaveric elbow joints obtained from adult males were selected (provided by the Teaching and Researching Section of Anatomy, Fuzhou Medical University, Fuzhou, China). The joints contained the upper and lower 2/3 of the diaphysis. The possibility of pathological changes of the sclerotin was ruled out by visual observation and X-ray scanning. The attachment of the triceps brachii tendon to the olecranon and joint capsule was preserved while the remaining soft tissues were removed. The samples were sealed in two-layer plastic bags and preserved in a refrigerator at −40°C. Prior to use, the samples were thawed at room temperature. A fretsaw with a diameter of 0.6 mm was used to amputate the bones which were made into three models: a transverse fracture model, an oblique fracture model and a comminuted fracture model which combined the two models (Fig. 1A). All treatment of cadavers was strictly in accordance with the international ethical guidelines and the National Institute of Health guide concerning the care and use of the human body. The experiments were approved by the Institutional human Care and Use Committee of the Fujian University of Traditional Chinese Medicine (Fuzhou, China).

#### Load and measurement

The samples were divided randomly into two groups: transverse fracture and oblique fracture models. Each group contained 10 samples. Five internal fixation techniques were applied to each fracture model (Fig. 1B–F). The samples were then made into comminuted fracture models and 10 samples were selected to test the five internal fixation techniques. The proximal end of the humerus was vertically fixed on the base of a WWL-100B electronic universal testing machine. A special clamping apparatus was used to hold and fix the aponeurosis of the triceps brachii and the elbow joint was flexed at 90°. The ulnar side was fixed to the clamping apparatus of the testing machine. A high-precision cantilever displacement sensor was placed at the bilateral sides of the olecranon fracture line where the tension was exerted. The sensor was connected to an SY-III digital strain meter. The load was applied level-by-level at a rate of 2 mm/min. The association between the load and displacement and the value of the tensile force when the fractured end was separated by 2 mm was recorded. Finally, two samples of the comminuted fracture which were fixed by each internal fixation technique were selected and a destructive load was applied. The relevant data were recorded.

#### Fixation materials

The materials used were 1.0-mm diameter wires, 2.0-mm diameter Kirschner needles and 3.5-mm diameter screws. An SY-III digital strain meter was used to apply the load at a constant speed and in a level-by-level manner. The association between the load and displacement and the tensile force value when the fractured end was separated by 2 mm was recorded. A destructive load was applied to the comminuted fracture model to test each internal fixation technique. The relevant data were recorded.

#### Surgical method

Brachiplex blocking anesthesia was administered and a pneumatic tourniquet was used to arrest bleeding. The affected limb was placed in front of the chest. A vertical incision was made at the center of the posterior cubital region, around the fracture site. The incision extended upwards and downwards for 3 and 4 cm, respectively, to expose the layers of tissues. The ulnar nerve was protected. The periosteum was cut open along the bony ridge and stripped to the bilateral sides to expose the fractured end and joint cavity. Hematoma and scar tissue were then removed. The fascia at the back of the olecranon was preserved as much as possible. The two ends of the fracture were repositioned and fixed with towel forceps. For old fractures, the triceps brachii was appropriately separated to reduce the tension so that the fracture was simple to reposition. For open fractures, thorough cleaning was necessary prior to the internal fixation. Next, a channel was drilled transversally at 1.5 to 2.0 cm away from the fracture line (equivalent to 1/2 of the olecranon thickness). Two wires 0.8 and 1.0 mm in diameter were passed through the channel. A wire with the ulnar side attached to the olecranon, was guided by a bone awl through the aponeurosis of the triceps branchii to the radial side. The wire thus formed a circle. The ulnar side of the other wire was guided from the back of the fracture to the radial side in an oblique manner. It also passed through the aponeurosis of the triceps brachii and reached the radial side from behind the fracture by attaching to the olecranon in an oblique manner. This wire formed a figure of eight configuration. When the fracture was repositioned, the circular wiring fixation was performed, followed by the figure of eight wiring. The free ends of the two wires were placed at the radial side of the distal end of the fracture and buried under the aponeurosis. The affected elbow was moved to examine its function and the condition of the articular surface. The wound was washed and stitched layer-by-layer. A rubber sheet was placed for drainage and removed the following day. Following the surgery, there was no need for external plaster fixation. Functional training began 2–3 days later.

#### Statistical analysis

Load-displacement curves were plotted for the three fracture models. A destructive load was applied to samples fixed by different internal fixation techniques to record the limit load. The SPSS 16.0 software was used for the statistical analyses. Variance analysis was performed on the means of multiple groups and if the variance analysis indicated significant differences, multiple comparisons were further employed. P<0.05 was considered to indicate statistically significant differences.

## Results

### 

#### Load and displacement

**Figure 2:**
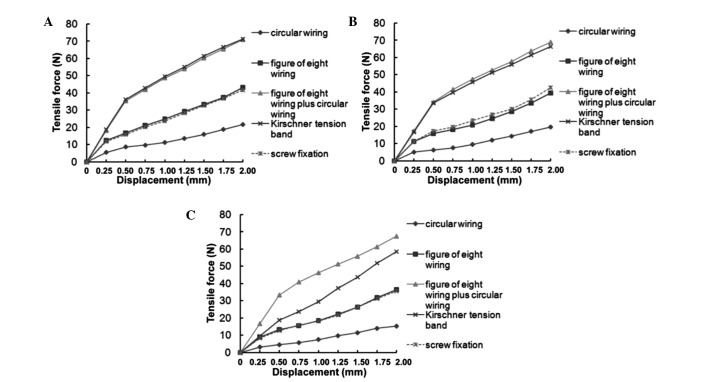
Correlation between load and displacement in the olecranon fracture models. The tensile force-displacement curve of (A) the transverse fracture model, (B) the oblique fracture model and (C) the comminuted fracture model.

The association between the load and displacement is shown in Fig. 2. The average tensile force values required for the separation of the fractured end by 2 mm in the three types of model using the five internal fixation techniques are shown in Table I.

Table I and Fig. 2 show that when elbow flexion reached 90°, the strain was essentially proportional to the load for all five fixation techniques. The circular wiring had the lowest fixation strength, while those of the figure of eight wiring and screw fixation were higher and similar to each other (P>0.05). The figure of eight plus circular wiring had a higher fixation strength than the circular wiring, figure of eight wiring or screw fixation (P<0.05). In the transverse and oblique fracture models, the figure of eight plus circular wiring exhibited no significant difference from the Kirschner tension band in terms of fixation strength (P>0.05). However, in the comminuted fracture model, the fixation strength of the figure of eight plus circular wiring was higher than that of the Kirschner tension band and the difference was significant (P<0.05).

#### Limit load

**Figure 3:**
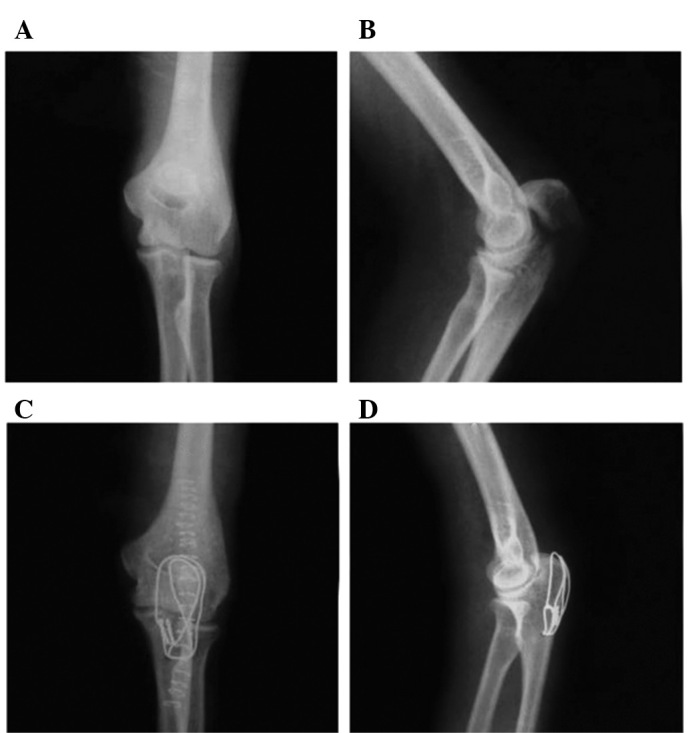
Representative X-ray films of olecranon fracture patients before and after treatment. (A) The positive X-ray film and (B) lateral X-ray film of olecranon fracture patients before treatment. (C) The positive X-ray film and (D) lateral X-ray film of olecranon fracture patients after treatment with figure of eight plus circular wiring, showing good positioning of the wires and an anatomical restoration of the fracture.

The values of tensile force required to damage the samples fixed using the five internal fixation techniques are shown in Table II and Figs. 3A and B. Table II shows that the Kirschner tension band had the largest limit load while circular wiring had the smallest. The figure of eight wiring and screw fixation had similar fixation strengths. The figure of eight plus circular wiring exhibited no significant differences from the Kirschner tension band (P>0.05). The absolute value of the limit load of the Kirschner tension band reached 84.5 N, higher than that of the figure of eight plus circular wiring.

### Clinical application

#### General data

A total of 152 olecranon fracture patients have been treated using figure of eight plus circular wiring internal fixation since 2001. The follow-up information was complete for 108 patients. The follow-up visits had an average duration of 12 months (Table III).

*Clinical assessment of therapeutic effect*X-ray images of the 108 patients revealed good positioning of the wires and the anatomical restoration of the fracture (Fig. 3). Follow-up visits, lasting for 12 months (range, 9–16 months), revealed the complete recovery of the fractures in all cases and the average recovery time was eight weeks. According to the Broberg scoring system (14), the recovery of 89 patients (82.4%) was excellent, 17 (15.7%)were fairly good and 2 (1.9%)were ordinary.

## Discussion

The olecranon is the endpoint of the triceps brachii muscle which is attached to the rear upper section of the olecranon. Following fracture, the proximal end becomes subject to the traction of the triceps brachii muscle and counteraction of the trochlea of the humerus, which generates pressure at the front of the olecranon and tension at the rear part. Therefore, the displacement of the fractured end was set as the experimental index. By referring to the method by Murphy *et al* (15), poor fixation was defined as the separation of the fractured end by 2 mm under load. According to the actual fixation position, load measurement was performed when the elbow was flexed at 90°.

Tables I and II and Fig. 2 show that the circular wiring had the lowest fixation strength, while figure of eight wiring and screw fixation had similar fixation strengths (P>0.05) and figure of eight plus circular wiring had a higher fixation strength than circular wiring, figure of eight wiring and screw fixation. The differences were statistically significant (P<0.05). In the transverse and oblique fracture models, the figure of eight plus circular wiring and the Kirschner tension band wiring exhibited no significant differences (P>0.05). However, in the comminuted fracture model, the fixation strength of eight plus circular wiring was significantly higher than that of the Kirschner tension band wiring (P<0.05). For the same fixation technique, the differences between the transverse and oblique fracture models were not significant (P>0.05). However, the difference was significant when compared with the comminuted fracture model (P<0.05). This observation indicated that the fixation stability of the transverse and oblique fracture models were not significantly different and the comminuted fracture model was less stable. The load curve of the circular wiring was the most flat in the three fracture models, without an apparent inflexion point. The load was linearly correlated with displacement. This finding suggested that this technique was unstable. In the transverse and oblique fracture models, both the screw fixation and figure of eight wiring had apparent inflexion points (S= 0.25 mm), corresponding to a load of approximately 18 N. The Kirschner tension band and figure of eight plus circular wiring both had inflexion points (S= 0.5 mm) in the transverse and oblique fracture models, corresponding to a load of 36 N. Beyond this point, the load was linearly correlated with displacement. This revealed that in the transverse and oblique fracture models, the four internal fixation techniques exhibited no separation of the fracture line before the load was increased to 18 or 36 N (S<0.25 or 0.5 mm, respectively). The fixation strength was similar. In the comminuted fracture model, only the Kirschner tension band and figure of eight plus circular wiring had apparent inflexion points. The difference was not significant before the displacement reached 0.5 mm. As the load increased, the figure of eight plus circular wiring had a higher fixation strength than the Kirschner tension band (P<0.05).

In the analysis of the experimental methods, there were three considerations: i) a 3-dimensional finite element model does not reflect the non-continuity of the fracture and the load of internal fixation; ii) a photoelastic test model does not fully simulate fracture models and the load of the internal fixation; and iii) the resistor disc used for measurement may not be easily fixed and is not suitable for the measurement of the displacement of the fracture end. Therefore, in the present study, we used a high-precision displacement sensor for a micro-study of the association between the load of an olecranon fracture and its stress. Simultaneously, a mechanical measurement method was performed to apply a destructive load. The new internal fixation technique was compared with four common internal fixation techniques: circular wiring, figure of eight wiring, Kirschner tension band and screw fixation. The purpose was to stimulate actual clinical models and to provide an experimental basis for the selection of appropriate internal fixation techniques clinically. Although the experimental data differed due to the variation in experimental conditions and methods, they all reflected the coherent mechanical characteristics of the commonly used internal fixation techniques. The circular wiring internal fixation had the lowest fixation strength, the figure of eight wiring and screw fixation were superior in terms of fixation strength and the figure of eight plus circular wiring and Kirschner tension band had the highest fixation strength. In the comminuted fracture model, the figure of eight plus circular wiring was the most stable.

Our long-term clinical observations also suggest that the figure of eight plus circular wiring internal fixation method has the following advantages for treating olecranon fractures: i) this technique conforms to the tension band principles and overcomes the shortcomings of screw fixation, such as a low bearing capacity, easy opening and separation at the rear; ii) a fixation wire is added to form a semi-spherical fixation structure, which significantly enhances the stability and resistance to tensile stress compared with figure of eight wiring; iii) this technique is easy to practice, overcoming the shortcomings of Kirschner tension band internal fixation of not being suitable for comminuted fractures and being difficult to adjust after fixation. It also prevents the tenderness around the pin site which may occur with the Kirschner tension band (16); iv) the technique requires less stripping and bleeding. It maintains the stability of the joint, and is less invasive, costly and time-consuming. Compared with hook plate internal fixation, it has a simplified procedure for internal fixation, thereby reducing the pain and economic burden for the patient; v) the technique makes use of the continuous force exerted by the aponeurosis of the triceps branchii, which is in accordance with the flexible fixation principle. It does not require drilling at the proximal end of the fracture and requires a smaller incision in the fascia of the olecranon for comminuted fractures. The technique makes use of the integrity of the aponeurosis of the triceps branchii for repositioning, making the surgery simpler to perform for comminuted fractures; and vi) during the removal, only a small cut under local anaesthesia is required to loosen and remove the wires. This process is simple to perform, thus hospitalization is not necessary for the removal of this internal fixation.

In conclusion, figure of eight plus circular wiring internal fixation may be applied to transverse, oblique and comminuted olecranon fractures. It appears to be a particularly good treatment for comminuted fractures. Characterized by low invasiveness, simple surgery, firm fixation and lower cost, this technique is safe, reliable and practical for internal fixation and should be popularized in clinical practice.

## Figures and Tables

**Table I:** Tensile force required for the separation of the fracture end by 2 mm.

		Tensile force, mean ± SD (N)				
Group	n	Circular wiring	Figure of eight wiring	Figure of eight plus circular wiring	Kirschner tension band	Screw
Transverse	6	21.72±1.54	43.21±1.82^a^	70.92±2.34^b,c^	71.40±2.16^b,d^	41.75±2.37^a^
Oblique	6	19.92±2.29	40.99±1.97^a^	70.26±2.16^b,c^	69.18±1.99^b,d^	42.63±1.80^a^
Comminuted	6	15.23±1.30	36.42±2.34^a^	67.42±2.17^b,c^	58.52±2.17^b,d^	35.73±3.23^a^

a P<0.05 compared with circular wiring;

b P<0.01 compared with circular wiring;

c P<0.05 compared with figure of eight wiring or screw fixation;

d P<0.05 compared with figure of eight wiring plus circular wiring.

**Table II:** Limit loads of the five internal fixation techniques.

Internal fixation technique	n	Limit load, mean ± SD (N)
Circular wiring	4	35.14±2.46
Figure of eight wiring	4	56.61±1.97^a^
Figure of eight plus circular wiring	4	81.43±2.61^b,c^
Kirschner tension band	4	84.45±2.52^b,c^
Screw fixation	4	57.62±2.91^a^

a P<0.05 compared with circular wiring;

b P<0.01 compared with circular wiring;

c P<0.05 compared with figure of eight wiring or screw fixation.

**Table III:** General olecranon fracture patient information.

Fracture type	Number of patients	Gender (male/female)	Affected limb (left/right)	Age, years, mean ± SD	Time interval between injury and surgery, days, mean ± SD	Duration of follow-up visit, months,mean ± SD
Transverse	35	18/17	15/20	38±7.6	3.4±1.2	12.3±1.94
Oblique	26	15/11	9/17	32±12.7	2.8±1.5	12.20±2.28
Comminuted	47	26/21	20/27	33±12.5	5.5±1.3	12.17±1.51
